# Cognitive behaviour treatment of co-occurring depression and generalised anxiety in routine clinical practice

**DOI:** 10.1371/journal.pone.0201226

**Published:** 2018-07-26

**Authors:** Roz Shafran, Abigail Wroe, Sasha Nagra, Eleni Pissaridou, Anna Coughtrey

**Affiliations:** 1 Population, Policy and Practice, University College London Great Ormond Street Institute of Child Health, Faculty of Population Health Sciences, London, United Kingdom; 2 Berkshire Healthcare NHS Foundation Trust, Berkshire, United Kingdom; Istituto Superiore Di Sanita, ITALY

## Abstract

**Background:**

Anxiety and depression are closely associated. However, they are typically treated separately and there is a dearth of information on tackling them together.

**Aims:**

The study’s purpose was to establish how best to treat co-occurring anxiety and depression in a routine clinical service—specifically, to compare cognitive behaviour therapy (CBT) focusing only on depression (CBT-D) to a broader CBT focusing on both depression and anxiety (CBT-DA).

**Method:**

Case notes of 69 patients with equally severe clinical levels of depression and anxiety seen in a routine clinical service were randomly selected to review from a pool of 990 patients. The mean age was 44.61 years (*SD* = 12.97). 65% of the sample were female and 88% reported their ethnicity white. The content of electronic records reporting techniques used and scores on a measure of depression (The Patient Health Questionnaire) and anxiety (The Generalized Anxiety Disorder Assessment) were reviewed to categorise therapy as CBT-D or CBT-DA.

**Results:**

Results indicated significant overall improvement with CBT; 70% and 77% of the sample met criteria for reliable improvement on The Patient Health Questionnaire and The Generalized Anxiety Disorder Assessment respectively. Fewer patients who received CBT-DA met The Generalized Anxiety Disorder Assessment recovery criteria at the end of treatment than those who received CBT-D. Mean post treatment PHQ-9 and GAD-7 scores remained above threshold for those receiving CBT_DA but not those receiving CBT-D. There was no evidence suggesting CBT-DA was superior to CBT-D.

**Conclusions:**

In patients with equally severe clinical levels of depression and anxiety, a broader treatment addressing both anxiety and depression does not appear to be associated with improved outcomes compared to treatment focused on depression.

## Introduction

Between 40% and 60% of patients with a common mental health disorder meet criteria for both anxiety and depression [1] [2]. These disorders share common risk factors, maintenance factors and respond to transdiagnostic interventions [3]. Changes in symptoms of anxiety and depression during and after psychological treatment are closely intertwined e.g., successful treatment of anxiety can result in improvements in depression [4]. Such is the closeness of their relationship that some researchers and clinicians have argued that they should be regarded as a single construct of ‘emotional disorder’ and that interventions should target neuroticism rather than separate categories of anxiety and depression [5].

Despite their close association, clinical guidelines and evidence-based psychological treatments have traditionally focused *either* on depression *or* a specific anxiety disorder [6] [7]. In the UK, guidelines recommend the use of cognitive behaviour therapy (CBT) as the first-line evidence based treatment for mild-moderate anxiety and depression [6] [7]. Challenges with such disorder-specific approaches in clinical samples where comorbidity is common have led to the development of protocolized ‘transdiagnostic’ interventions such as those of Barlow and colleagues [8]. Such approaches appear efficacious in a range of formats [3] [9] and confer clear advantages over multiple disorder specific approaches in terms of cost-effectiveness and ease of dissemination [10].

Taking a transdiagnostic treatment approach is only one of a number of options available to clinicians in routine clinical practice who face the daily challenge of addressing anxiety and depression in their patients in the absence of research data and guidelines to inform their decision-making. Despite the merit of such approaches and interest in them, the quality of existing research studies is relatively poor and certainly lags behind those evaluating disorder-specific approaches in which many clinicians will have been trained [3].

Alternative approaches to the problem of comorbidity are to use evidence based disorder-specific interventions to (i) focus on the treatment of one of the disorders and measure the outcomes in both, or (ii) address both disorders simultaneously, sequentially or alternating between them. There are relatively little data on each of these treatment options. One relevant study is that of Craske and colleagues in which sixty-five patients with panic disorder and a comorbid anxiety disorder were randomly assigned to CBT focused solely upon panic disorder or CBT that simultaneously addressed panic disorder and the most severe comorbid condition [11]. Results indicated that those receiving CBT focused only on panic disorder were more likely to meet high end-state functioning at post-treatment and zero panic attacks at the one-year follow-up. It was concluded that remaining focused on CBT for panic disorder may be a better treatment option both for the primary and comorbid diagnoses than combining CBT for multiple disorders.

A similar finding, that it is better to stay focused on one disorder rather than addressing multiple disorders, was obtained by Gibbons and deRubeis [12]. In this study, 24 patients with both anxiety and depression participating in a CBT for depression trial were found to have a worse outcome for both depression and anxiety if the therapist addressed both disorders rather than remaining focused on depression. There is a paucity of other studies directly speaking to this important issue but for patients with major depressive disorder, it does not appear that broader, arguably complex cognitive therapy is superior to the more focused intervention of behavioural activation [13] and behavioural activation may even have advantages in severe cases [14].

What do clinicians choose to do in routine clinical practice when confronted with patients who have common comorbidities such as anxiety and depression which are of equal severity? The answer is not known for certain due to the lack of data on this vital clinical question. The indications are that the majority of clinicians in routine clinical practice, such as the UK’s Improving Access to Psychological Therapies (IAPT) services, do not use disorder-specific interventions designated as efficacious but instead prioritise clinical judgement to provide an ‘eclectic’ approach [15] [16] [17].

In summary, there is very little information on the best way to address co-occurring anxiety and depression in routine clinical settings. The aim of this study was to compare the clinical outcome of CBT delivered in a routine clinical setting (an IAPT service) on depression and anxiety when CBT focused on one of these disorders vs CBT that focused on both anxiety and depression. In the process of conducting the study, it quickly became apparent that insufficient numbers of therapists had focused on CBT addressing anxiety alone to allow a valid comparison of the approaches; the present study was therefore only able to compare CBT focused on depression alone (CBT-D) with CBT focused on both anxiety and depression (CBT-DA). CBT-D was defined as CBT which only included components of Beck’s CBT for depression protocol (e.g. cognitive restructuring and behavioural activation; [18] [19] whereas CBT-DA included components of both Beck’s CBT for depression protocol and techniques from Dugas’ treatment of anxiety disorders such as tolerating uncertainty [20] [21].

Based primarily on the studies of Craske et al. [11] and [12], it was hypothesised that patients with clinical levels of anxiety and depression who received CBT focused only on depression would have a better clinical outcome for both depression and anxiety than those receiving two interventions.

## Method

The authors have abided by the Ethical Principles of Psychologists and Code of Conduct as set out by the APA at http://www.apa.org/ethics/code/. The project was approved by the Royal Holloway University Ethics Board (ref 07/15). All data were anonymised and part of the routine IAPT data collection.

### Design

The study was a retrospective case-note review. The data had already been collected as part of routine clinical management and was stored on the patient management and reporting tool used as part of the UK’s national IAPT service ‘IAPTus’. Therapists are required to enter the minimum dataset (MDS) every session which includes measures of depression (The Patient Health Questionnaire; PHQ-9 [22]) and anxiety (The Generalized Anxiety Disorder Assessment; GAD-7 [23]). They are also required to enter case notes from each session which includes details of the nature and content of each therapeutic contact e.g. purpose of the appointment, the primary and secondary interventions used, a summary of the content of the appointment and treatment plan.

### Setting

We reviewed cases from all IAPT sites in a specific NHS Foundation Trust. All IAPT therapists have received training in CBT protocols for anxiety and depression, received regular supervision and are skilled clinicians (the majority were employed at NHS Agenda for Change Band 7), which means that they are typically within five years of qualification. The cases reviewed were treated by 44 different clinicians.

### Measures

#### PHQ-9

The PHQ-9 is a well-established 9-item measure assessing depression symptoms. Scores range from 0 to 27, with a clinical cut off of 10 and above.

#### GAD-7

The GAD-7 is a well-established 7-item measure assessing GAD (Generalised Anxiety Disorder) and other anxiety disorder symptoms. Scores range from 0 to 21, with a clinical cut off of 8 and above.

Also collected as part of the MDS for each session was Work and Social Adjustment Scale scores [24], IAPT Phobia Scale scores and IAPT Employment Questionnaire responses, as well as disorder specific questionnaires when applicable [25].

Both the PHQ-9 and GAD-7 are patient reported outcome measures which are administered by the therapist who uses the scales collaboratively with the patient to monitor progress for clinical purposes of tracking outcome. Both measures are completed by the patient every session and reviewed by the therapist, and are designed so that even the least experienced clinicians would be able to detect high levels of both anxiety and depression in their clients based on these measures. Both the PHQ-9 and GAD-7 are standardised measures widely used in clinical practice. Both have well established psychometric properties including demonstrated diagnostic validity when compared to structured clinical interview (e.g. [26] [23]).

#### Session content proforma

Based on the treatment protocols recommended by NICE [6] [7], the Competence Frameworks for anxiety and depression ([27] and the IAPT Training Curriculum [25], a proforma was developed to assess the content of each session. Techniques used to address components of the depression and anxiety protocols were each noted on the proforma. This proforma checklist was developed, reviewed and adjusted by the frequency of use of each technique in each session, as well as qualitative notes for ‘other’ treatment researchers using the 18 pilot participants.

The final version of the proforma included background information i.e. presenting problems, treatment goals and the plan for therapy. The summary of the treatment received was based on the presence of the following techniques: worry awareness/usefulness, coping with uncertainty, imaginal exposure (Dugas protocol components); and problem solving, behavioural activation, coverage of core beliefs/rules/dysfunctional assumptions, cognitive restructuring (Beck protocol components). Calculations were then made regarding the numbers of times, in all therapy sessions for each person, the following were used: generic anxiety or worry techniques, Dugas treatment manual components, Beck key components, other depression approaches or techniques and other techniques (e.g. post-traumatic stress disorder).

### Case selection

#### Sample size calculation

It was difficult to conduct a power analysis as this is the first study of its type. However, based on previous similar studies [11] [12] a power calculation indicated that approximately 60 cases would be sufficient to detect a small-moderate effect size (*d =* .25) at 80% power with *p* < .05 for CBT focussed on depression vs. CBT focused on anxiety and depression. Previous research has found a significant difference between groups with 24 patients [12] and a related study found significant differences using 65 patients [11]. It was conservatively estimated that approximately 50% of cases reviewed would meet inclusion criteria, therefore we aimed to review 135 cases using the session content proforma.

#### Inclusion criteria

Case notes were deemed eligible to review for the study based on the following inclusion criteria. Firstly, the patient must have received at least 6 sessions of one-to-one ‘High Intensity’ traditional CBT delivered face-to-face. A minimum of 6 sessions was chosen as less than 6 sessions is what is commonly delivered as a brief ‘low-intensity’ intervention in routine clinical practice [28] [29]. Additionally, the primary problem needed to be recorded as ‘depression’, ‘recurrent depression’, ‘GAD’ or ‘mixed depression and anxiety’. Although ‘mixed anxiety and depression’ in DSM-IV was intended to be used for patients who are subthreshold on anxiety and depression diagnostic criteria but have clinical symptoms of both [30], it is often used within IAPT to indicate the presence of both anxiety and depression [28]. The primary problem was typically recorded as anxiety if the patient reported symptoms of generalised anxiety disorder such as worry. If the primary problem was recorded as a specific other anxiety disorder such as social anxiety, they were not included.

Further, the patients were required to meet ‘clinical caseness’ for anxiety and depression according to the PHQ-9 and GAD-7 questionnaires. Caseness is defined as scoring 10 or above on the PHQ-9 and 8 or above on the GAD-7. Given concerns over the use of the primary problem descriptor label ‘mixed anxiety and depressive disorder’ within IAPT services, caseness on the PHQ-9 and GAD-7 was used in addition to relevant problem descriptor categories. The criteria of ‘caseness’ has been used in previous analysis of IAPT data (e.g. [28]).

Finally, it was necessary that cases were scored with equal levels of severity on measures of depression and anxiety at the start of treatment. This was to ensure that the cases represented those in clinical practice where it was unclear which disorder to focus on because they were of equal severity and one was not primary. For the purpose of this study cases were considered ‘moderate’ if they scored were between 10 and 19 on the PHQ-9, and between 8 and 14 on the GAD-7. Cases were considered ‘severe’ if they scored between 20 and 27 on the PHQ-9 and between 15 and 21 on the GAD-7. This was based on standard IAPT severity index scores [25].

#### Case review

**Fig 1:**
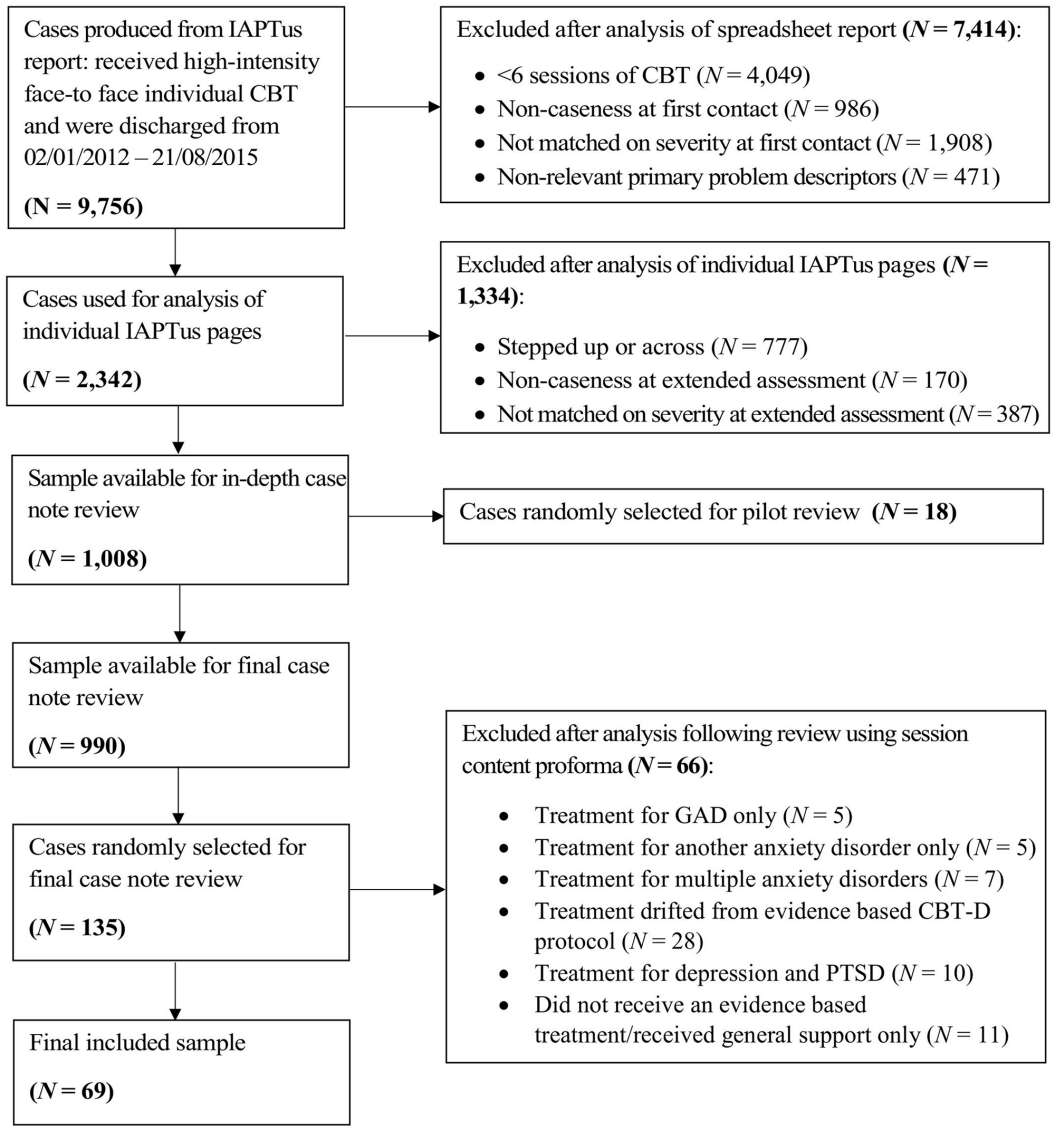
Selection process and exclusion criteria for the case review.

9,756 patients were seen in the IAPT service of the NHS Foundation Trust reviewed between 02/01/2012 and 21/08/2015. As can be seen in Fig 1, this gave a pool of 1,008 cases eligible to review. 18 of these were selected at random using the “= RAND()”randomise function in Microsoft Excel to conduct a pilot review (detailed below) to develop the proforma. 135 cases from all sites were selected at random from the remaining sample for the final case note review using the same method. These were then reviewed using the session content proforma and a final 69 cases were selected for analysis. There were too few participants who received CBT focused on anxiety to include in the study so the analyses focused on those who received CBT for depression (CBT-D) and those that received CBT for depression and anxiety (CBT-DA). There were no significant differences in medication use, number of sessions, symptom severity, PHQ-9 or GAD-7 scores between cases included and excluded in the final analysis (all *p*’s > .05).

### Inter-rater checks

10 cases of the 135 were selected at random and classified independently by the authors as to whether the intervention was coded as ‘Anxiety only’, ‘Depression only’, ‘Depression and Anxiety’ or ‘Other’. There was 100% agreement among the raters.

### Participant characteristics

Participants ranged from 20 to 70 years old (*M* = 44.61, *SD* = 12.97). 65.22% of participants were female, 30.43% disclosed long term health condition(s) and 56.52% reported some form of previous mental health treatment. At the first session 68.12% of participants stated they were prescribed and taking psychotropic medication, 63.77% reported being employed full or part time, and 11.6% reported they were receiving statutory sick pay at the time. PHQ-9 scores at session one ranged from 10 to 27 (*M* = 18.58, *SD* = 4.91). GAD-7 scores at session one ranged from 8 to 21 (*M* = 15.28, *SD* = 3.93).

### Focus of intervention

Of the 69 cases included for analysis, 30 received an intervention that focused on depression only and 39 received an intervention focused on depression and anxiety. 32 out of 39 receiving CBT-DA received both treatments simultaneously, and only 7 received treatments sequentially, therefore sequence of treatment was not examined in the outcome analysis. The number of treatment sessions varied between 6 and 21.

### Reliable recovery

A patient was considered to have “recovered” if they have dropped below the clinical cut offs on the PHQ-9 and GAD-7, based on Jacobson and Truax’s [31] criteria and IAPT standard index severity scores [25]. A “reliable change” index was also used to establish whether patients made a reliable improvement, no change, or reliable deterioration. According to Jacobson and Truax’s [31] reliable change criteria and previous studies [28], in order for patients to have made a reliable improvement in depression symptoms their PHQ-9 scores must have reduced by at least 6 points since baseline, and GAD-7 scores by at least 4 points since baseline for anxiety symptoms. Patients were considered to have made a reliable improvement if scores reliably reduced on both measures. The final index of “reliable recovery” was used to measure whether or not a patient has met both previous criteria. A patient was considered to have made reliable recovery if their last clinical outcome measures showed both reliable improvement and fell below the cut off for clinical caseness.

### Data analysis

The primary outcome measures were the PHQ-9 and GAD-7. Analyses were based on the reliable recovery index (as defined above) and on repeated PHQ-9 and GAD-7 measurements of 69 patients. Logistic regression models were fitted to the data to assess the association between reliable recovery and treatment. Reliable recovery was considered as a dichotomous outcome. Linear mixed effects models [32] were fitted to the longitudinal data allowing for random effects for individuals and serial correlation amongst the repeated PHQ-9 and GAD-7 measurements using restricted maximum likelihood. A linear trend in scores over time was assumed and adjustments were made for gender and baseline measurements. Descriptive statistics and parametric and non-parametric tests are given as appropriate. Data was collated using IBM SPSS 22. Statistical analysis was undertaken using R (version 3.3.2) and the R package nlme [33] [34].

## Results

### Overall clinical outcome

Scores on the baseline PHQ-9 and GAD-7 were significantly correlated (*r* = .84, *p* < .001). Mean pre and post questionnaire scores are given in Table 1. Paired t-tests were applied to examine the mean difference in PHQ-9 and GAD-7 scores (see Table 1). The differences between pre-treatment and post-treatment scores were significant (PHQ-9 *p* < .001 and GAD-7 *p* < .001) suggesting that participants showed a significant reduction in depression and anxiety symptoms by the end of treatment. Table 2 also shows that outcomes met IAPT national targets for recovery and are consistent with previous studies of IAPT outcomes [28].

**Table 1:** Means, standard deviations and t-test statistics for pre and post PHQ-9 and GAD-7 scores.

	Pre			Post			Mean difference	*t*	*df*	*p*
	*N*	*Mean*	*SD*	*N*	*Mean*	*SD*				
PHQ-9	69	18.58	4.91	69	9.84	6.40	8.74	11.96	68	0.000*
GAD-7	69	15.28	3.93	69	8.04	5.61	7.23	10.92	68	0.000*

*p < .001

**Table 2:** Percentage of reliable improvement, reliable recovery and recovery by treatment.

	Single depression	Multiple depression and anxiety
Reliable improvement (%)	70.00	61.54
Reliable improvement on PHQ-9 (%)	76.67	64.10
Reliable improvement on GAD-7 (%)	76.67	76.92
Reliable recovery (%)	60.00	41.03
Reliable recovery on PHQ-9 (%)	63.33	53.85
Reliable recovery on GAD-7 (%)	66.67	46.15
Recovery (%)	66.67	46.15
Recovery on PHQ-9 (%)	66.67	58.97
Recovery on GAD-7 (%)	70.00	46.15

### Reliable recovery

The odds of reliable recovery on PHQ-9 for patients receiving multiple depression and anxiety treatment were .63 times the odds of reliable recovery on single depression treatment (adjusted OR 0.63; 95% CI [0.22, 1.74]; *p* = .38). Similarly, CBT-DA was associated with a 62% decrease in the odds of reliable recovery on GAD-7 compared to CBT-D (adjusted OR 0.38; 95% CI [0.13, 1.05]; *p* = .07). These associations were not significant. Adjustment was made for gender and severity.

### Comparing CBT-D vs. CBT-DA

The non-parametric Mann-Whitney U test indicated there were no significant differences in the distribution of baseline PHQ-9 and GAD-7 scores across the two treatment groups (*p* > .05) (descriptive statistics are given in Table 3). There was a significant difference in age at time of treatment between the groups (independent samples t-test *p* = .026) with those who received treatment for depression only being older than participants who received treatment for both depression and anxiety. However, age was not correlated to change in the PHQ-9 (*r* = .08, *p* = .53) or GAD-7, (*r* = .13, *p* = .29). Chi-squared tests did not suggest that gender, severity, medication and long term conditions are associated with treatment group with *p*-values of .83, .55, .89 and .55 respectively.

**Table 3:** Means, standard deviations of questionnaires at session one.

	Depression only						Depression and anxiety					
	*N*	Mean	Median	*SD*	Min	Max	*N*	Mean	Median	*SD*	Min	Max
Pre-PHQ-9	30	19.37	20.00	4.97	11	27	39	17.97	18.00	4.84	10	26
Pre GAD-7	30	15.17	16.00	3.92	9	21	39	15.36	14.00	3.98	8	21

The mean post treatment PHQ-9 score for participants receiving CBT-DA (*M* = 10.18, 95% CI [8.11, 12.24]) was above the clinical cut off of 10, whereas the mean for participants receiving CBT-D (*M* = 9.40, 95% CI [6.96, 11.84]) was below threshold. The same was true for GAD-7 scores, with the mean post score for participants receiving CBT-DA (*M* = 8.64, 95% CI [6.77, 10.51]) being above the clinical cut off of 8, and below the cut off for participants receiving CBT-D (*M* = 7.27, 95% CI [5.25, 9.29]). However, the confidence intervals cross the clinical cut off points.

Non-parametric independent-samples Mann-Whitney U tests were run for the number of sessions to recovery. These indicated no significant differences in the number of sessions to recovery between the two treatment groups (number of sessions to recovery on PHQ-9: CBT-D *M* = 6.13, CBT-DA *M* = 5.57, *p* = .29, number of sessions to recovery on GAD-7: CBT-D *M* = 6.04, CBT-DA *M* = 6.16 *p* = .91 and number of sessions to recovery on both: CBT-D *M* = 6.83, CBT-DA *M* = 6.76, *p* = .66). These analyses indicate that participants in both groups took an equal number of sessions to recover based on their questionnaire.

The interaction between treatment and time (session) in the linear mixed effects model for PHQ-9 was significant (estimated coefficient 0.29; 95% CI [0.02, 0.58]; *p* = .04). This indicates that the association between treatment group and PHQ-9 score depends on time, that is, with more sessions, greater differences in PHQ-9 scores between the two groups are expected. The average difference in PHQ-9 scores between CBT-DA at session 7 and CBT-D at session 7 was 2.31 (estimated difference 2.31; 95% CI [0.04, 4.57]; *p* = .05).

There was no evidence to suggest that the association between treatment group and GAD-7 score depends on time at the 5% significance level (estimated interaction between treatment group and time 0.24; 95% CI [-0.06, 0.53]; *p* = .12). There was no difference in GAD-7 scores for CBT-DA compared to CBT-D at session 7 (estimated difference 1.53; 95% CI [-0.62, 3.68]; *p* = .16).

## Discussion

The findings indicated that both symptoms of anxiety and depression decrease significantly when treated with both CBT focused on depression and CBT targeting both depression and anxiety. Approximately two-thirds of patients showed reliable improvement and recovery from their symptoms of depression, and three-quarters showed reliable improvement on symptoms of anxiety although only 56% could be considered ‘recovered’ on this measure. There were relatively few significant differences between the two groups. There were other indications of the possible superiority of CBT-D compared to CBT-DA in that the mean depression and anxiety scores for participants receiving CBT-DA but not CBT-D remained above the clinical thresholds post-treatment but these differences were not significant with the current sample size. In addition, the mean post treatment depression and anxiety scores for participants receiving CBT-DA were above the clinical thresholds whereas the mean for participants receiving CBT-D were below threshold.

Overall, there was no evidence to suggest that CBT-DA is superior to CBT-D.

The study goes some way to answering the research question posed with regard to optimal approaches to the treatment of comorbid anxiety and depression although it is noteworthy that the question of clinical outcome when CBT focuses on anxiety only could not be answered since there were too few cases. There are several possibilities for why clinicians would prefer to choose treatment that includes depression in cases of comorbidity. First and foremost, clinicians may be reluctant to treat patients with depression with an anxiety protocol due to concern about risk. Second, clinicians may feel more competent and comfortable with CBT for depression rather than CBT for anxiety. Most clinicians in routine psychological services are first trained in CBT for depression and then trained in specific anxiety disorder protocols. Perhaps the order in which clinicians are trained influences the order in which the treatments are provided in cases of ambiguity and comorbidity. It is possible that clinician experience influences decision making, with more experienced clinicians viewing CBT-D as a simpler and equally effective approach, leading to more effective treatment in this group. Alternatively, it may be the case that some of the clinicians observe that anxiety improves when depression is successfully addressed and therefore see no reason to include CBT for anxiety as an additional component. One reason for this observed improvement may be due to the overlap in symptoms between anxiety and depression (e.g. [35], [36]); it would be interesting for future work to investigate whether CBT-DA does have an added benefit for specific symptoms of anxiety. Another explanation is that CBT protocols for anxiety are more specified (and arguably more prescriptive) than Beck’s protocol for depression which allows other difficulties to be addressed within it e.g. managing anxiety, sleep. It is therefore possible that treatment for depression is more likely to involve additional treatment for anxiety, whereas treatment for GAD is less likely to involve additional treatment for depression. It may also be that ‘therapist drift’ [17] is more likely to occur in CBT-D as our review of 135 case notes indicated that 28 drifted from the evidence-based protocol and a further 10 incorporated techniques from CBT for Post Traumatic Stress Disorder. Further research is needed to explore these issues and to understand therapist decision making and their appraisals of their patients. It is likely that therapists will use clinical judgement to identify patients for whom they think a combination is likely to be more effective. For example, they may believe that more complex patients require CBT-DA in order to address the range of symptoms, or alternatively they may make formulation based judgements as to why a particular patient may not respond to a combination treatment. Understanding the decision making process is an important area for future research as it would both capture clinician wisdom and provide data on how clinicians incorporate research evidence into everyday practice.

It could be argued that the clinicians who used both depression and anxiety protocols did so in order to engage their clients and reduce drop-out. It was not possible to identify the drop-out rates as planned session numbers were not recorded in the case notes. However, the range of sessions reflects the recommended number of sessions as recommended by NICE [6] [7] (2009, 2011) and there was no difference in the mean number of treatment sessions received by the groups, suggesting that those receiving CBT-D were equally engaged in treatment as those receiving CBT-DA. This is not surprising since it does appear that patients are engaged with, and form strong therapeutic alliances with, therapists when their symptoms are improving (e.g. [37]). An alternative explanation is that focusing on depression only (rather than a comparison treatment) may lead to expectancy effects in patients, whereby they are more likely to expect symptom change and subsequently report symptom reduction to please the therapist.

This study adds to the small literature addressing a big clinical question. However, the study is limited by its design—it was not a randomized controlled trial but a retrospective case note review with a relatively small sample although the sample was selected at random which increases the likelihood of representativeness. Nevertheless, there are limitations to making post-hoc assessments about presenting psychopathology from case notes and it is possible that the treatment content was not accurately reported by therapists. In addition, the validity and reliability of the proforma was not independently established. Future research should be prospective and incorporate video or audio review of treatment sessions. It is possible that the presence of factors such as use of psychotropic medication confounded the results and this should be explored in future research, perhaps using large public datasets. In addition, the high correlation between the PHQ-9 and GAD-7 at baseline may account for the effectiveness of CBT for depression in cases of comorbidity. Further research with additional measures of anxiety and depression are required to explore this issue further.

Despite its limitation, this study and the existing literature do not suggest that outcomes for either anxiety or depression are improved by the addition of CBT for anxiety to CBT for depression in cases of comorbidity. This is consistent with the more general finding that severe and complex presentations with multiple co-existing disorders do not appear to benefit from more complex interventions. The finding appears counter-intuitive but it may be that a simple, focused intervention provides patients (and therapists) with a clear sense of understanding about what needs to happen to change, mastery and control whereas multiple methods, suggestions, ideas and techniques can be overwhelming. Until future prospective studies are conducted to address such questions, this research is a good starting point as it contributes to the understanding of single and combined treatments, that doing one intervention may well be better than trying to provide multiple interventions.
